# Tracking the Unconscious Generation of Free Decisions Using UItra-High Field fMRI

**DOI:** 10.1371/journal.pone.0021612

**Published:** 2011-06-27

**Authors:** Stefan Bode, Anna Hanxi He, Chun Siong Soon, Robert Trampel, Robert Turner, John-Dylan Haynes

**Affiliations:** 1 Max Planck Institute for Human Cognitive and Brain Sciences, Leipzig, Germany; 2 Department of Neurology, Otto-von-Guericke University Magdeburg, Magdeburg Germany; 3 Psychological Sciences, The University of Melbourne, Melbourne, Australia; 4 Melbourne Medical School, The University of Melbourne, Melbourne, Australia; 5 Bernstein Center for Computational Neuroscience Berlin and Charité – Universitätsmedizin Berlin, Berlin, Germany; 6 Neuroscience and Behavioral Disorders, Duke-NUS Graduate Medical School, Singapore, Singapore; 7 Graduate School of Mind and Brain, Humboldt Universität zu Berlin, Berlin, Germany; University College London, United Kingdom

## Abstract

Recently, we demonstrated using functional magnetic resonance imaging (fMRI) that the outcome of free decisions can be decoded from brain activity several seconds before reaching conscious awareness. Activity patterns in anterior frontopolar cortex (BA 10) were temporally the first to carry intention-related information and thus a candidate region for the unconscious generation of free decisions. In the present study, the original paradigm was replicated and multivariate pattern classification was applied to functional images of frontopolar cortex, acquired using ultra-high field fMRI at 7 Tesla. Here, we show that predictive activity patterns recorded before a decision was made became increasingly stable with increasing temporal proximity to the time point of the conscious decision. Furthermore, detailed questionnaires exploring subjects' thoughts before and during the decision confirmed that decisions were made spontaneously and subjects were unaware of the evolution of their decision outcomes. These results give further evidence that FPC stands at the top of the prefrontal executive hierarchy in the unconscious generation of free decisions.

## Introduction

As humans, we experience the ability to consciously choose our actions as well as the time at which we perform them. It has been postulated, however, that this subjective experience of freedom may be no more than an illusion [1], [2] and even our goals and motivations can operate outside of our consciousness [3]. Early studies discovered the presence of a so-called readiness-potential (or Bereitschafts-potential, BP), a slow negative potential shift in EEG activity that precedes voluntary movement [4]. Libet et al. demonstrated that the readiness-potential also precedes the conscious awareness of initiating the movement by several hundred milliseconds, and was therefore attributed to unconscious processes preceding voluntary actions [5]. The readiness-potential was believed to originate from the SMA/pre-SMA and the anterior cingulate motor areas [6], [7]. There is indeed some evidence that the SMA/pre-SMA is involved in focusing on self-initiated actions and performing self-initiated movements [1], [8]–[13].

The early studies by Libet et al. have been criticized for several reasons. It has been questioned whether the timing judgments were reliable, given that the time-window between reported intention and movement was short (about half a second) and attention to aspects of timing could cause additional distortions [14]–[17]. Because the original study only investigated the readiness potential from movement-related brain regions, it is further possible that pre-SMA/SMA might only be involved in late stages of motor planning, while other higher cognitive control regions might be more likely candidates for planning the decision outcome [18]–[20]. Furthermore, it is important to investigate whether any choice-predictive signals reflect an unspecific, global preparation of a decision or whether they are related to the specific outcome of a decision, as suggested in subsequent work based on lateralized readiness potentials [21].

In recent years, the combination of multivariate pattern classification with functional magnetic resonance imaging (fMRI) has yielded a novel way to address this problem [22]–[26]. These new approaches have previously been used to investigate the encoding of the content of decisions and abstract task rules [27]–[29] and indeed have already given new insights into the realm of free decisions. In a recent study, participants were asked to freely and spontaneously decide to press either a left or right button at their own pace [30]. A visually presented continuous letter stream, refreshed every 500 ms, served as a clock, which participants used to indicate the time at which they first became aware of their intention to press the left or right button. Multivariate pattern classification was applied to the fMRI data from discrete time-bins *preceding* each decision to search for information encoding throughout the whole brain. This approach made it possible to resolve the major pitfalls of previous studies: First, this approach allowed the investigation of brain activity many seconds prior to a decision. Second, rather than searching for regions with unspecific activation changes, it can be used to find regions that encode the specific content of an intention. It was possible to decode the decision outcomes of such free motor decisions from the pole of anterior medial prefrontal cortex (BA 10) and the precuneus/posterior cingulate cortex (PCC), up to 7 s before subjects were aware of their intention [30]. The results clearly pointed to frontopolar cortex (FPC) as one possible site of origin for free decisions. The decision outcome was encoded in fine-grained spatial activation patterns that were not detectable using more conventional univariate analyses.

Here we replicated the original study using ultra-high field fMRI on a 7-Tesla scanner, which allowed us to acquire images with 1×1×1 mm^3^ voxels. Specifically, we were interested in the role of frontopolar cortex and thus only brain images from anterior FPC were recorded, which allowed a higher spatial resolution of the target region and a better temporal resolution of the early components of the decision making process. These improvements allowed us to explicitly investigate the temporal stability of these early decision-related patterns, which was not addressed in the original study [30]. Furthermore, after the scanning session we assessed our subjects' behaviour and their thoughts during the experiment to investigate factors that may have biased the decision outcomes. This provided evidence in determining whether early predictive activity patterns already reflect conscious aspects of the decision process or whether these are related to truly unconscious components of evolving intentions.

## Materials and Methods

### Ethics statement

The study was approved by the local ethics committee at the Max Planck Institute for Human Cognitive and Brain Sciences, Leipzig, Germany. The study was carried out in accordance to the Declaration of Helsinki. Informed written consent was obtained from each subject before the study.

### Participants

Twelve right-handed subjects (5 female, average age 24 years, age range 22–29 years) participated in the fMRI experiment. All subjects were students of the University of Leipzig, enrolled in various fields of study. All were healthy and had normal or corrected to normal vision, and had no history of neurological disease. Suitable subjects were selected by means of behavioural pre-tests, conducted within the 2 weeks preceding the fMRI session. These pre-tests consisted of 5 blocks of the same task as used for the fMRI experiment and ensured that only those subjects who *inherently* fulfilled important criteria were selected for the fMRI session. First, the frequency with which a subject chose each of the two possible outcomes (left button or right button) needed to be balanced, meaning that one option should not have been chosen more than twice as often as the other. Second, we selected subjects that “naturally” performed trials at a moderate pace (i.e., at a speed of 15 to 50 seconds per trial). This pace allowed an optimal separation of fMRI signals for different trials. These first two criteria were not known to the subjects such that they had maximal freedom in their decisions, but it was specifically emphasised that their decisions should be unbiased and spontaneous. Third, based on post-experimental questionnaires it was ensured that subjects made *spontaneous* decisions and did not pre-plan them. The behavioural performance from the fMRI session was evaluated using the same criteria. We did not pre-select subjects according to their level of intelligence or any other cognitive capacity. Data from one subject (S4) was discarded from all analyses due to relatively unbalanced decisions and exceptionally long trial durations. Data from one run of another subject (S12) had to be discarded due to technical problems with recording buttons.

### Experimental Paradigm

**Figure 1:**
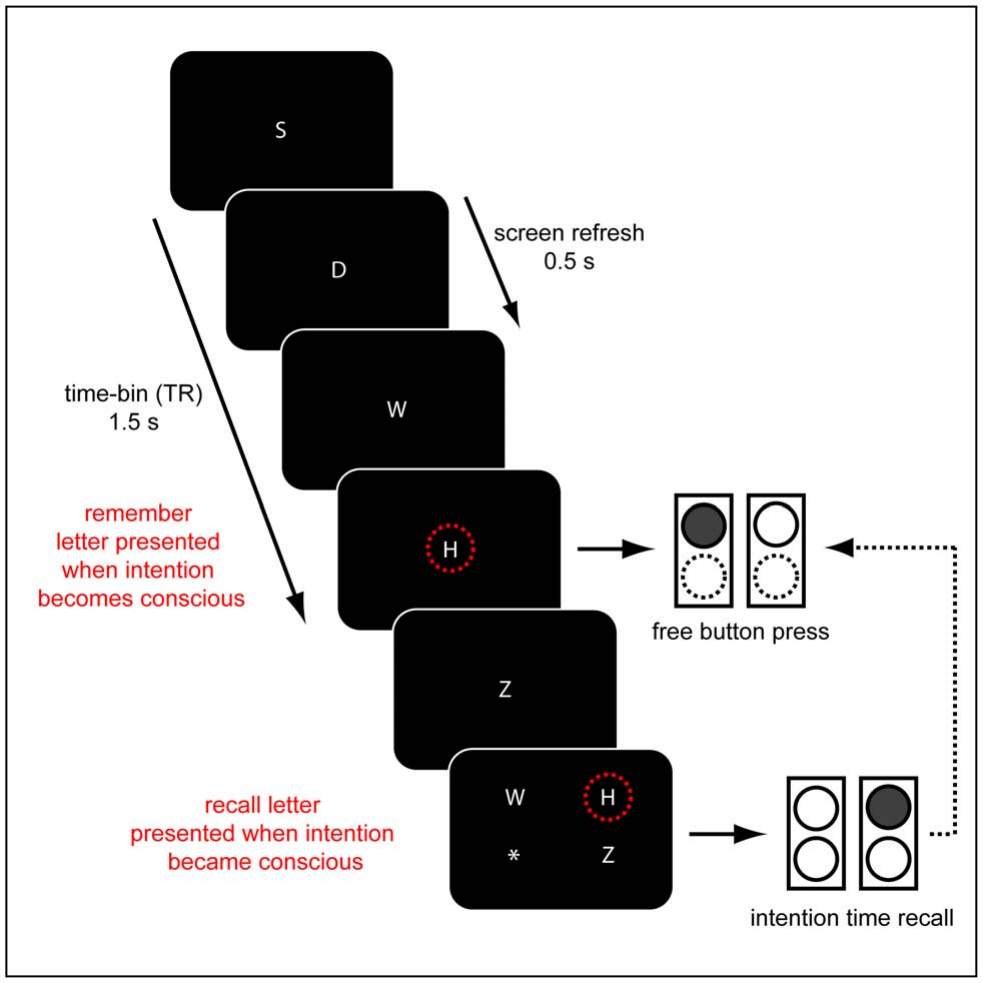
Experimental paradigm. Subjects were presented with a stream of constantly changing white letters on dark background. The screen was refreshed every 500 ms. The task was to freely and spontaneously decide to press a response button with the left or the right index finger (illustrated by upper circles; decision for left button in example illustrated by filled circle) whenever they felt the urge to do so. They were instructed to note the letter displayed on the screen when they became aware of their intention and to immediately perform the button press (the letter H in the example; red circles are for illustration and were not presented). Following the button press, a response screen was presented. Three letters and an asterisk were presented in the four corners of the screen, the letters being those shown during and immediately prior to the button press. Subjects indicated the letter that was visible at the time of the decision by pressing the button corresponding to its position on screen (recalled letter H, indicated by upper right button in example). If they could not remember the letter or if the relevant letter was not present, they indicated this with the asterisk. After the response was given, the next trial started and subjects were instructed to return to a relaxed state before making a new decision. The general paradigm was taken from Soon et al. [30].

A stream of stimulus screens was presented at a rate of 2 Hz, (refreshed every 500 ms). Each stimulus frame displayed a central letter on a dark background. Only consonants were used and were presented in a pseudo-randomised order such that the same letter never occurred twice within 8 consecutive frames. Subjects were instructed to passively view the stream of letters, relax, and refrain from thinking about the upcoming task. The index- and middle fingers of both hands rested on 4 buttons of two joysticks. Subjects were free to decide, at any time, to press the left or the right button with the corresponding index finger. As soon as they were aware of their decision, subjects were to note the letter presented on the screen. The time at which subjects are first aware of their decision will hereafter be referred to as the “decision time” in short. Subjects were instructed to then immediately perform the chosen action without any delay. Once a button was pressed with the index finger, a response screen was presented for a variable delay of 1, 1.5, 2 or 2.5 s. This screen contained three letters and an asterisk arranged in a square. Subjects were then to indicate the letter noted at the decision time by pressing the corresponding button on the joystick, the four buttons now corresponding to the position of the letters (or asterisk) on the screen. The three letters always corresponded to the three letters shown immediately prior to and including the button-press (0 s, −0.5 s and −1 s relative to the button press). Their positions were also randomized. If subjects were unable to recall the letter present at the time of the button press, or if the relevant letter was not displayed, they were told to select the asterisk (see Fig. 1). Using a letter stream as a timing device allowed us to detect whether decisions were planned ahead of time (see [30]). Upon completion of the trial, the stream of stimuli resumed. Subjects again relaxed and passively viewed the stream of stimuli until the next decision was spontaneously made. Note that subjects were only instructed to relax and not to pre-plan their decisions at any time; the pacing and the ratio of left and right decisions were deliberately left to them in order to avoid any artificial restrictions of the free decision process. As described above, pre-tests were used to select only those subjects for the fMRI session who inherently showed optimal behavioural performance.

After the scanning session, subjects completed a questionnaire about their subjective experiences with the experiment. They were asked to report their thoughts and behaviour during the experiment, even if these had contradicted the task instructions. Participants were asked to rate on a five-point scale (0 = *never* to 4 = *always*) I) how often they made a decision earlier during the trial but waited before executing the button press; II) their spontaneity throughout the experiment (0 = *not spontaneous at all* to 4 = *very spontaneous*); III) how often they explicitly thought about the timing of their decisions (0 = *never* to 4 = *always*). Additionally, they had to describe IV) the content of their thoughts between trials, and V) if they noticed any changes in their performance during the experiment.

### Functional Imaging

**Figure 2:**
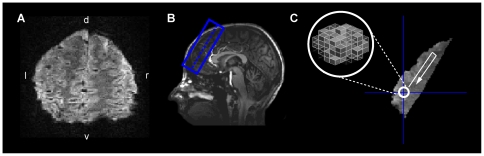
Illustration of EPI image, slice positioning and decoding approach. A) Example of one slice of one participant's EPI image. B) Structural T1 image from the same subject displaying the positioning of the example slice (dotted line) and slice coverage (blue box). For each subject, 21–25 coronal slices (1×1×1 mm^3^, without gap) were positioned such that the most anterior part of frontopolar cortex was covered. Due to the optimized slice positioning, which allowed the use of a small field of view (FOV) and a short echo train length, a relative small part of the air-filled cavities was included. This improved the quality of the EPIs and reduced signal dropouts and distortions. C) The parameter estimates from the FIR model were used for multivariate pattern classification. A moving “searchlight” algorithm was implemented using a radius of 3 voxels in order to decode the outcome of the upcoming decision from each position in frontopolar cortex.

Functional imaging data was acquired using a 7-Tesla whole-body MR scanner (MAGNETOM 7T; Siemens, Germany) with an 8 channel array head coil (RAPID Biomedical, Rimpar, Germany). A gradient echo planar imaging (EPI) sequence was used for functional imaging (TR = 1500 ms, TE = 23 ms, flip angle = 90°, matrix size 64×64, in-plane resolution 1×1 mm). 21–28 slices were acquired (1 mm thickness, no gap), depending on the SAR limit of individual subjects, and covered the most anterior part of prefrontal cortex. In order to minimise signal dropout due to the frontal sinuses, the slices were tilted away from the coronal orientation by an angle of 30.2° to the transverse plane (due to the anatomy of individual subjects and their position in the scanner, the angle was 37.6° in two cases and 36.0° in one case). Particularly for ultra-high field strength, signal dropout and distortions in frontopolar cortex can be substantial. This setup was found to maximally reduce signal distortions and dropouts for the present study because it allowed us to use a small field of view (FOV), and thus a short echo train length, in order to cover most of anterior prefrontal cortex with maximal exclusion of the air-filled cavities compared to axial slices (Fig. 2). However, using this setup no region beyond frontopolar cortex could be covered. Data was acquired for 10 functional runs, each lasting 5 minutes (200 volumes per run). The first two volumes of each run were discarded by default to allow for magnetic saturation effects. Additionally, a structural T_1_-weighted image was acquired for each subject for co-registration (176 transversal slices; TR = 3840 ms, TE = 268 ms; voxel resolution 1×1×1 mm^3^). During the scanning sessions, stimuli were presented via a projector with a resolution of 1024×768 pixels (refresh rate of 60 Hz) that projected from behind the head-end of the scanner onto a screen. Subjects lay supine in the scanner and viewed the projection via a mirror. Responses were recorded using a set of two custom-engineered, deconstructed Nintendo Wii joysticks, each with two buttons operated with the index- and middle fingers of either hand.

### Multivariate Pattern Analysis

Multivariate pattern classification was used to analyse the data. The analysis sought to identify regions within FPC that allowed subjects' decisions for left and right to be decoded from fine-grained patterns of activity as measured by the BOLD signal *preceding* the subjects' conscious awareness of their decisions. Chance level for correct prediction was 50%.

The first stage of data processing involved motion correction to the first image of the first run using SPM2 (http://www.fil.ion.ucl.ac.uk/spm/). No additional normalization or smoothing was performed at that stage in order to maximize the sensitivity for information encoded in the fine-grained voxel patterns [24], [31], [32]. A finite impulse response (FIR) predictor was used to model fMRI responses, as it was not known whether the profile of the fMRI time course adheres to the generic haemodynamic response function in this situation. This procedure also allowed time-resolved decoding to be implemented [29], [30]. Left-button trials and right-button trials were modelled as two separate conditions, each with 20 FIR regressors. Each regressor modelled a time-bin of 1.5 s (1 TR), covering a 30-second time period around each trial. The 10^th^ time-bin was defined as that in which a decision was made. The first 10 regressors therefore modelled the 15 seconds *preceding* (and including) each decision, the last 10 covered the 15 seconds *following* the decision. Invalid trials (in which subjects were unable to recall a letter) were modelled separately, again using 20 FIR predictors and assigning the 10^th^ predictor as that in which the button was pressed. These trials were excluded from the pattern classification analyses. To minimise unaccounted-for variance in the fMRI data, the second button-presses with which subjects indicated the letter present at the decision time were modelled as covariates. Left-handed and right-handed button-presses were modelled separately, and convolved with a standard haemodynamic response function (HRF).

In the next step, a “searchlight” decoding procedure was applied to the data of each subject separately, using the activity patterns of different local spherical voxel clusters [26], [27], [29], [30]. This way the choice-predictive information encoded in a spherical cluster of voxels at each position in the brain can be estimated without making any a-priori assumptions as to the location of the information (Fig. 2C). As a direct replication of Soon et al. [30], the searchlight radius was kept at r = 3 voxels. Note that in the original study the voxel size was 3×3×3 mm^3^ while the reduction of voxel size in the present study to 1×1×1 mm^3^ yielded a searchlight 27 times smaller. Nevertheless, information decoding was based on the same number of data points (voxels) in both cases. The total number of voxels in the whole search volume in anterior PFC was comparable to that in a whole-brain data set with the standard 3×3×3 mm^3^ voxel resolution acquired on a 3-T scanner. For every voxel in the volume (denoted by v_i_), a spherical searchlight cluster of N voxels was defined around it, such that all voxels in the cluster (denoted by c_(1…N)_) lay within a radius of 3 voxels of v_i_. For every voxel in c_(1…N)_, the parameter estimates for all 20 time-bins were extracted from each run, separately from left-decision and right-decision trials. These were transformed into two N-dimensional pattern vectors (one corresponding to left-decision trials, the other to right-decision trials) for each of the 20 time-bins, representing the spatial activation patterns for both decisions at all 20 points in time. To assess how much discriminative intention-related information was contained in these patterns at each time-point, pattern vectors (each time-point separately) from nine of the ten runs (eight of the nine runs for the subject with one excluded run) were first assigned to a “training data set”. This set was used to train a linear support vector machine (SVM) pattern classifier (LIBSVM implementation, http://www.csie.ntu.edu.tw/~cjlin/libsvm) to discriminate between patterns corresponding to the two different decision outcomes (or intentional states), using a fixed regularization parameter C = 1 [33]. The classifier estimated a decision boundary separating the two classes of patterns in N-dimensional space where N is the number of voxels in the local spherical cluster (also see [34]). The amount of intention-related information contained in these patterns was then assessed by using the decision boundary to classify the trials in the independent “test data set”, taken from the remaining run, as belonging to the left-decision or right-decision condition. This procedure was repeated 10 times, each time using a different run as the independent test data set, resulting in a 10-fold cross-validation. The pattern classification results from each repetition were averaged and assigned to the central voxel of the searchlight cluster as its decoding accuracy.

The entire procedure was repeated by assigning in turn every voxel in the brain volume as the central voxel of the searchlight cluster, yielding a 3D map of decoding accuracies throughout the imaged volume. Furthermore, such a 3D decoding accuracy map was obtained for each of the 20 time-points. These maps represent the amount of intention-related information encoded in the local neural networks at each location in the brain (of each individual subject), at the time-point from which the parameter estimates were taken. In the next step, the subjects' individual decoding accuracy maps were normalized to MNI-space. For this, the functional images were first co-registered to the individual high-resolution T_1_-weighted structural whole-brain image, which was acquired during the same scanning session. The T_1_-weighted image was normalized to the MNI T_1_-template image as implemented in SPM2. The normalization parameters were then applied to the decoding accuracy maps. These were further smoothed with a Gaussian kernel of 3 mm FWHM. Voxels that were not shared by all subjects were masked out. For each time-point, group level analyses were performed across subjects. The decoding accuracy maps from the time-bins *preceding* the decision (time-bins 1–10) were analysed, yielding a statistical parametric map of voxel clusters (using a 5 voxel threshold) that displayed decoding accuracies greater than chance level (50% for two decisions) during the 15 seconds preceding (and up to) the conscious decision time (using a threshold of *p*<.05 FDR corrected). It was therefore possible to track changes in the amount of information encoded in different regions over time, and in particular, to search for a build-up of intention-related information prior to subjects' conscious awareness of their own intentions, as observed by Soon et al. [30].

### Temporal pattern stability

**Figure 3:**
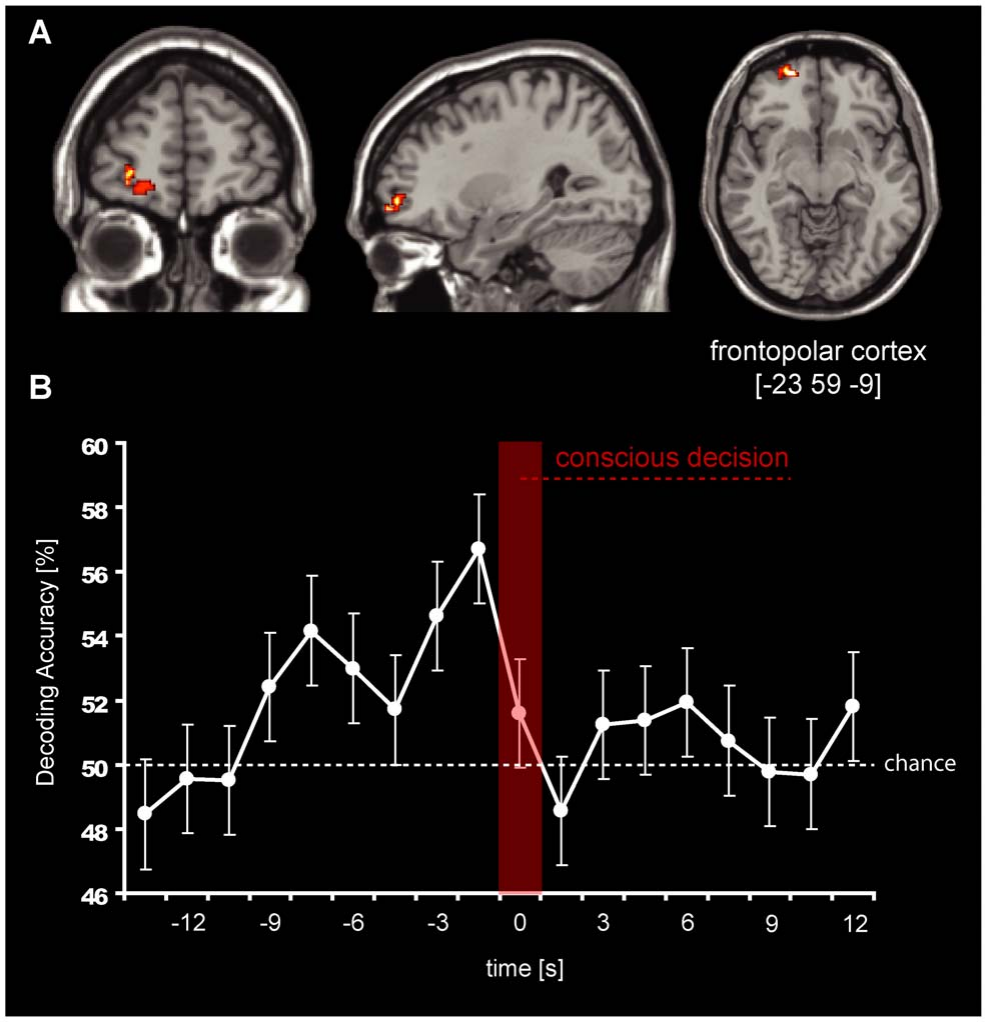
Decoding of upcoming motor decisions from frontopolar cortex. A) The figure displays a region in left frontopolar cortex [−23 59 −9] from which decoding was possible significantly above chance (50%) using a threshold of *p*<.05 (FDR corrected; voxel-threshold 5 voxels). FPC only showed significant decoding accuracies in the time-bins preceding the decision. B) The graph displays the average time-course of decoding accuracies, taken from the central voxel of the searchlight cluster that showed the highest decoding accuracy. Error bars represent standard errors. The time-bin of the conscious intention is indicated by the red bar and is labelled as time 0. Time-points preceding the conscious awareness of the intention are labelled as negative numbers (units = seconds, relative to decision); time-points following the decision are therefore positive. One time-bin corresponds to 1.5 s. Coordinates displayed are MNI coordinates.

The goal of this analysis was to investigate the spatial-temporal profile [35] of the time-bins that allowed the prediction of free decisions before they reach conscious awareness. Individual data from the searchlight yielding the highest decoding accuracy across subjects (see Results and Fig. 3A) preceding the decision was analysed across time-bins for each subject. For this, this coordinate, which was established after normalizing the individual 3-dimensional decoding accuracy maps, was transformed back into individual space. The spherical cluster with radius r = 3 voxels was again constructed around this position and the spatial activation patterns for each decision were extracted and transformed into pattern vectors, separately for each run and individually for each subject.

The pattern vectors from single time-bins were then combined for each decision by I) simply *averaging* vectors in steps of (i) two, (ii) three or (iii) four time-bins or by II) *concatenating* vectors in the same steps. The multivariate pattern classification analysis was run again on these new vectors, exactly as described before; the difference was that there were a smaller number of time steps per analysis. If averaging across earlier time-bins does not reduce the decoding accuracy, this would mean the spatial activation patterns display a consistently high temporal stability in those time-bins. Finally, correlation analyses were also conducted between the pattern vectors of adjacent time-bins (separately for each subject and each condition), in order to assess the temporal stability of these patterns in more detail.

Since the results did not differ for left and right decisions, the results are reported for each time-bin for all the analyses. Please note that all these subsequent analyses only aimed to specify the role of the best searchlight cluster and not to select voxels for further dependent statistical analyses, which would have been circular [36]. Also note that the chosen cluster was the best decoding cluster *averaged across* subjects. This cluster therefore did not represent the optimal decoding cluster in individual subjects. Analysing the optimal clusters in individual subjects, however, would have carried the risk of arbitrariness and was therefore strictly avoided.

### Univariate Control Analysis

The parameter estimates obtained from a GLM, based on normalised and smoothed (3 mm FWHM) data, were used in a conventional mass-univariate analysis. Again, a finite impulse response (FIR) predictor was used to model fMRI responses (identical to the analysis described above) and group level analyses were performed across subjects for each time-point separately. The purpose of this analysis was to investigate whether any voxels at any time-point showed significant differences in activation between the left-decision and right-decision trials.

## Results

### Behavioural Results

Subjects chose the left button on 51% of all trials, and the right button 49% of all trials. There were only very few trials in which subjects could not recall the letter present when their decision was made (average 1% of all trials). In nearly all trials, subjects indicated that the decision reached conscious awareness during the presentation of the same letter or one letter before they pressed the button (see Table 1 for details). Our criterion was that any subject who showed highly unbalanced decisions (criterion: one option was not chosen more than twice as often as the other), or had exceptionally short or long trial durations (criterion: on average <15 s or >50 s) were excluded from further analyses. One subject (S4) was thus excluded. For nine of the remaining eleven subjects, the individual probability of left and right decisions did not significantly differ. Only two subjects tended to make more left than right decisions (S1: *χ^2^*(1) = 4.36; S2: *χ^2^*(1) = 5.63; both *p*<.05) In total, the average time lapsed between consecutive trials was 29.7 s (SD = 9.29); an average of 11 trials was performed per 5 min run. This was even longer compared to the original study [30]; spill-over effects from the previous trial therefore can not explain the results.

**Table 1:** Behavioural results.

subject	left [%]	right [%]	mean trial duration (SD) [s]	letter indicated [mean]	“*” indicated [%]
S1	56%	44%	20.7 (7.8)	−0.19	1
S2	59%	41%	23.2 (5.5)	−0.17	0
S3	46%	54%	24.0 (6.3)	−0.13	1
*S4*	*39%*	*61%*	*58.4 (20.4)*	*−0.11*	*7*
S5	50%	50%	46.4 (8.2)	−0.17	0
S6	56%	44%	42.4 (8.0)	−0.09	0
S7	50%	50%	26.8 (6.2)	−0.13	0
S8	43%	57%	28.1 (7.1)	−0.12	0
S9	50%	50%	36.2 (14.7)	−0.07	4
S10	55%	45%	24.4 (8.9)	−0.13	2
S11	45%	55%	36.6 (15.0)	−0.93	5
S12	52%	48%	18.0 (4.0)	−1.36	2

Note: left/right = percentage of left/right decisions across experiment; mean trial duration given for each subject in seconds; “letter indicated” refers to the number of letters lapsed between the time at which a decision became conscious and the time at which the button was pressed (one letter = 500 ms duration). On average, all subjects indicated that the intention reached conscious awareness during the same time-bin (500 ms) or one time-bin earlier relative to the button press (0 = same time-bin, −1 = preceding time bin); Subjects indicated by pressing the “*” that they did not remember the letter presented when they became aware of their decision.

In the post-experimental interviews subjects indicated that they were able to relax and make spontaneous decisions. They reported to rarely wait with the execution of the button press after being aware of their intentions (scale from 0 = *never* to 4 = *always*; M = 0.9; SD = 0.54), as instructed. They indicated having been very spontaneous (M = 3.3; SD = 0.65) and they did not pay much attention to the timing (M = 1.0; SD = 0.89). Most subjects reported that they did not have specific thoughts they could remember. Some reported having thought about (or mentally read) the letters, some reported having occasionally thought about daily activities but none reported having thought about the decisions. Most subjects reported that they became more relaxed through the experiment and that they either became more spontaneous or that there was no change in spontaneity. This is not surprising given that subjects were highly familiar with the task, having completed 10 runs of prior training, and were able to perform the task effortlessly. Comparable to the original study [30], the distribution of sequence lengths (periods of the same decision before a switch occurred) resembled an exponential distribution, as expected for random behaviour. Furthermore, we correlated the sequences of decisions from each run of each subject with the sequence of decisions in the following run, in order to control for the possibility that subjects might have simply repeated fixed sequences of decisions over the experiment. None of these correlations were significant for any of the subjects (all tests *p*>.05; average correlation *r* = −.11; range −.27 to .07). For each subject and within each functional run, we further analysed whether the sequences of left and right decisions violated the assumption of a random order (runs test as implemented in MATLAB, MathWorks Inc., corrected for multiple tests). We note, however, that due to the nature of our task, the number of successive trials per functional run were very short, thus, this test has limited informative value. The results showed that out of all 110 functional runs from all 11 subjects, violations of the randomness assumption could only be found in one single run (*p* = .004; all others *p*>.05), providing additional evidence that behaviour was spontaneous. These results indicate that participants performed correctly and that preplanning or other unaccounted-for activity cannot explain the decoding results (see Table 2 for individual results).

**Table 2:** Individual post-experimental interview results.

subject	Quest. I	Quest. II	Quest. III	Quest. IV	Quest. V
S1	2	4	2	the day, girlfriend, relaxing	nothing
S2	1	4	1	letters, nothing	more relaxed
S3	1	3	3	nothing	more relaxed
*S4*	*excluded*	*-*	*-*	*-*	*-*
S5	1	3	1	nothing	nothing
S6	1	3	0	nothing	nothing
S7	1	3	0	nothing	decisions slightly faster
S8	1	3	1	letters	more relaxed, spontaneous
S9	0	4	1	letters, uni, holidays	forgot letters few trials
S10	1	2	1	letters	nothing
S11	0	3	0	nothing	decisions slighty slower
S12	1	4	1	nothing	Nothing

Note: Question I: “How often did you make a decision earlier during the trial but waited with the button press?” (0 = never; 4 = always); Question II: “How would you rate your spontaneity throughout the experiment?” (0 = not spontaneous at all; 4 = very spontaneous); Question III: “How often did you explicitly think about the timing of your decisions?” (0 = never; 4 = always); Question IV: “What did you think about during the experiment, as far as you can remember?”; Question V: “Did you notice any changes in your behaviour during the experiment? If yes, what changed?” One subject (S4) was excluded from all analyses because of behavioural criteria.

### Brain Imaging Results

**Figure 4:**
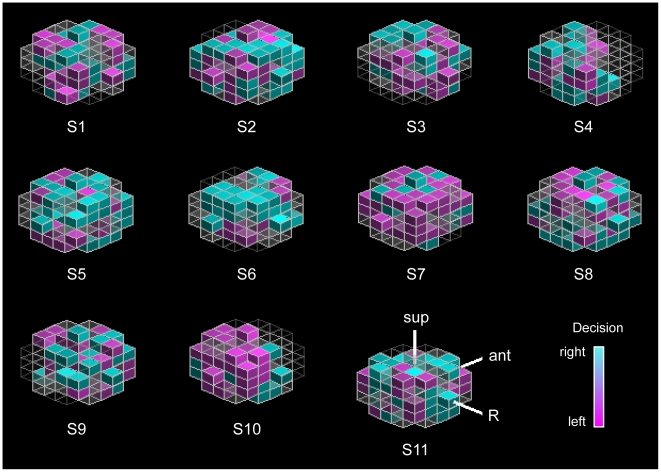
Individual searchlight clusters. Displayed are the spherical voxel clusters (with radius r = 3 voxels) in frontopolar cortex of all subject that yielded the highest decoding accuracy in the time-bin directly preceding the decision (−1.5 s). Voxels responding preferentially to one decision are colour-coded (magenta for left, aqua for right; sup = superior, ant = anterior, R = right). Grey transparent voxels did not show decision preference or were not located in grey matter. Colours are scaled for better visualization. Informative patterns were different for each participant.

Multivariate pattern classification analysis was used to search for brain regions encoding subjects' decision outcomes. We identified a cluster in FPC from which subjects' decisions could be decoded *before* their intentions became conscious (i.e. time-bin 10, Fig. 3), with statistically significant decoding accuracies of up to 57% (SE = 1.69; *p*<.05 FDR-corrected) just before the decision was made (time-bin 9; see Fig. 3). This region was located in left frontopolar cortex (MNI coordinates −23 59 −9; see Fig. 4 for individual searchlight clusters). The earliest time at which decoding was possible was ∼7.5 seconds (time-bin 4) before the decision was reported to be consciously made. Taking into account the temporal delay of the BOLD signal (which is in the order of a few seconds), it is possible that these signals reflect processes up to 10 seconds before the actual decision.

**Figure 5:**
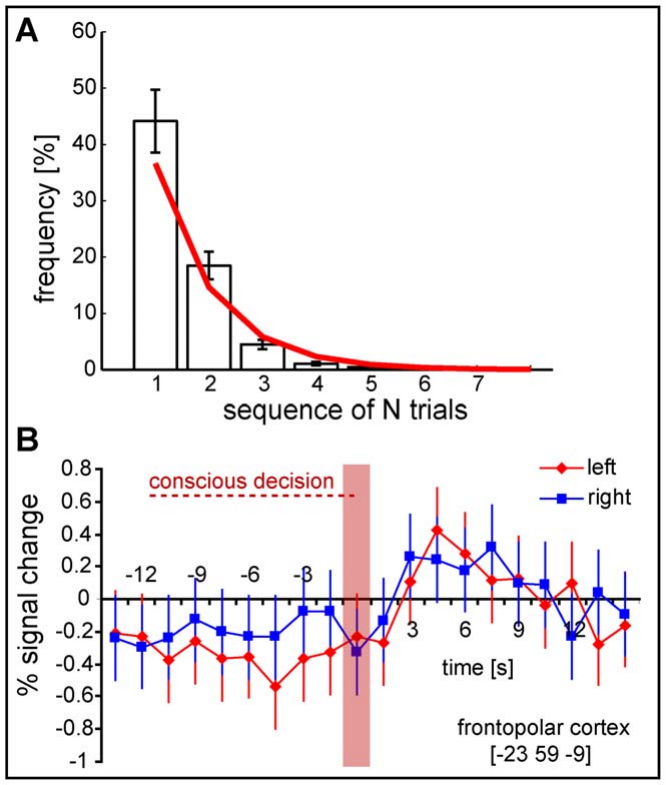
Sequence length and univariate fMRI results. A) Histogram of sequence length. Displayed is the average percentage of sequences of N trials of the same decision (left or right) before switching to the other decision. It resembles an exponential distribution; fitted model: f(x) = 100*c*e^−c*x^, with c = 0.917, RMSD = 2.739 (red curve). This suggests that subjects made random decisions. Error bars are standard errors. B) The graph shows the percent signal change (average BOLD estimates from 20 FIR predictors) during left-decision and right-decision trials for the central searchlight voxel [−23 59 −9] that demonstrated the highest accuracy in decoding the decision outcome prior to the conscious decision. For both conditions, the signal increased only *after* the decision (red bar) and came back down to baseline in the next ten seconds. Significant differences between left and right decisions were not found for this cluster. No region could be found in the imaged volume that displayed a difference between left and right, even when a liberal threshold of *p*<.001 (uncorrected) was used. Time-points preceding the conscious awareness of the intention are labelled as negative numbers (units = seconds, relative to decision); time-points following the decision are therefore positive. One time-bin corresponds to 1.5 s. Coordinates are given as MNI coordinates.

Using a searchlight radius of r = 4 voxels led to decreased decoding accuracies and *p*-values for the same region. When the radius was further increased, no significant results could be achieved, possibly due to the increased dimensionality. In a control analysis the accuracy maps from the time-bins *after* the decision was made (time-bins 11–20) were contrasted against chance level. No clusters could be found in FPC encoding any information above chance level during this period. The same held true if separate time-bins around the time of the motor response were considered, matching the findings from the original study that FPC only encoded the intentions *before* subjects were aware of making a decision. The information was, as in the original study, only encoded in fine-grained activation patterns rather than in the average signal. Additional univariate analyses confirmed that there was no significant difference between left and right decisions at any time in individual voxels, even when a liberal threshold of *p*<.001 (uncorrected) was applied. This held true for the whole frontopolar region as well as for the region from which decoding was possible. The average BOLD signal did not increase at all until after the decision was made (see Fig. 5B).

**Figure 6:**
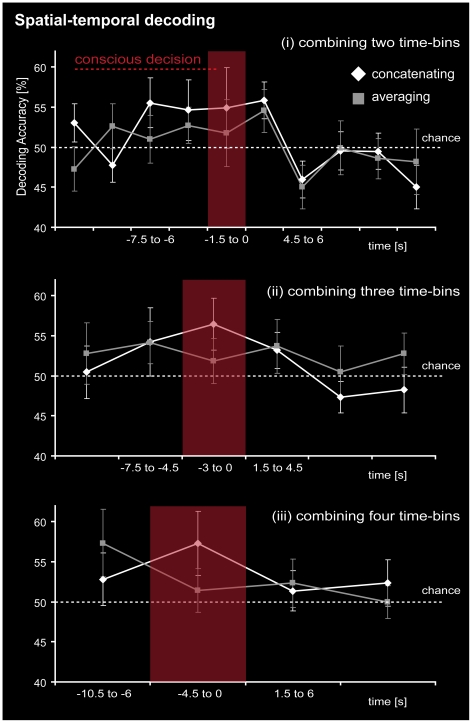
Temporal pattern stability. Temporal-spatial decoding analysis. The spatial activation patterns from the searchlight cluster, which was found to give best results in decoding accuracy (MNI −23 59 −9), was extracted in the individual subjects' data. The original patterns from the time-bins (1 time-bin = 1.5 s) were combined by concatenating (white) and averaging (grey) the respective pattern vectors in steps of either (i) two time-bins, (ii) three time-bins, or (iii) four time-bins. The reference time-bin for vector concatenation was the time point of the decision (time 0 s). The resulting pattern vectors additionally represented temporal information for the best searchlight cluster and were used for multivariate decoding. Temporal-spatial information was found to be highest directly preceding the decision and was still present when four time bins were concatenated. Concatenating was superior to averaging by trend.

**Figure 7:**
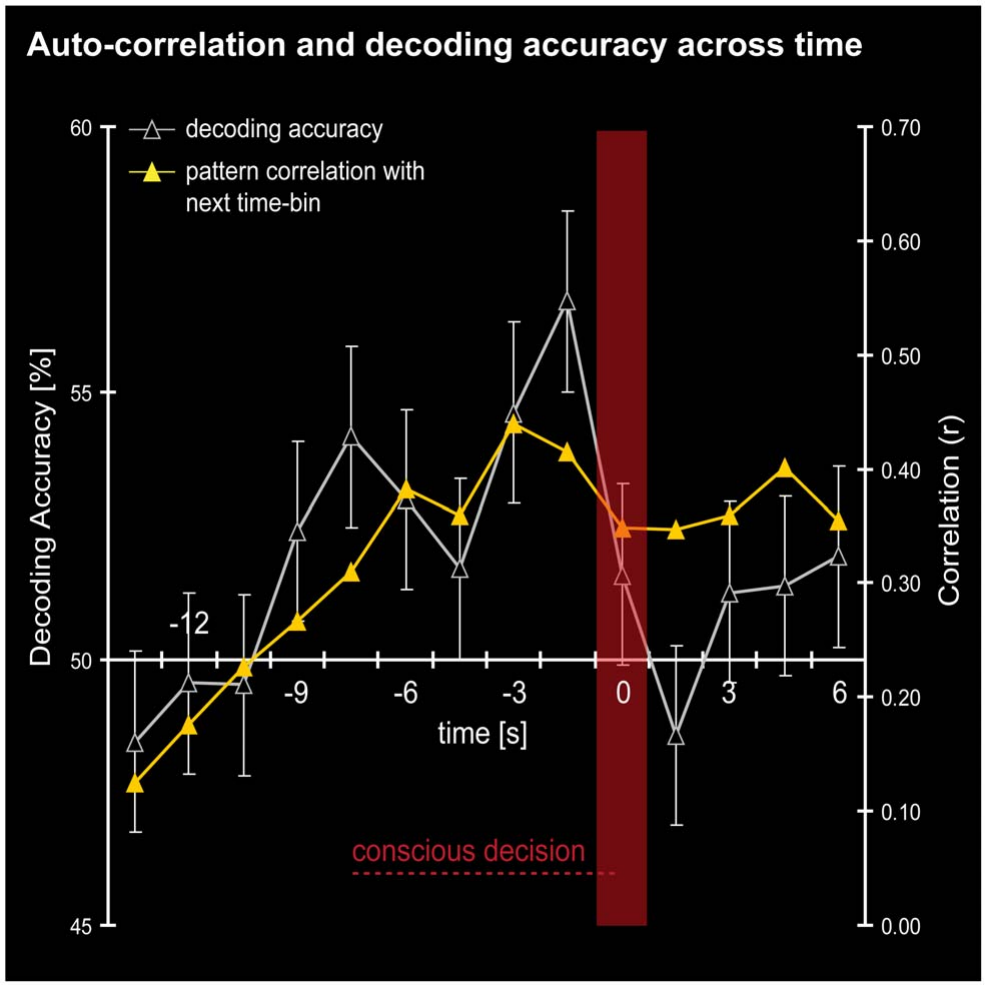
Correlation analysis for spatial activation patterns. Displayed is the decoding accuracy across time from the best cluster (empty gray triangles) as well as the correlation of each time-bin with its preceding time-bin (filled yellow triangles) as a measure of pattern similarity (averaged across patterns for left and right decisions). Up to the time of the decision (time 0 s) the decoding accuracy and pattern similarity increased in a similar fashion. After the decision, the pattern similarity dropped slightly and patterns did not predict the decision outcome anymore.

The subsequent decoding analysis using the best searchlight cluster across all subjects showed that averaging across adjacent time-bins led to lower decoding accuracies, the more time-bins that were combined (Fig. 6). Concatenating time-bins, which is combining spatial and temporal information before the decision, still predicted the decision outcome with high accuracy (Fig. 6). Concatenating was superior to averaging by trend, which suggests that, in the time leading up to a decision, spatial patterns were not uniform throughout, but carried more decision-related information with increasing temporal proximity to the decision. In subsequent correlation analyses we found that patterns from consecutive time-bins nevertheless showed significant correlation. Moreover, there was increasing pattern similarity with increasing temporal proximity to the decision (peak *r* = .44; *p*<.05; Fig. 7). After the time-point of the decision, the correlations dropped again to a stable level. This auto-correlation curve closely mimicked the time-course of decoding accuracies (Fig. 7). Thus, activity patterns became more similar and more informative the closer the decision-maker was to becoming aware of the decision. After the decision was made, some pattern stability was sustained but the patterns no longer carried information about the decision.

## Discussion

This study aimed to assess whether local spatial activity patterns in FPC, which were previously found to encode unconscious intentions [30], display temporal stability over time. Using ultra-high field fMRI at 7T, allowing a voxel resolution of 1×1×1 mm^3^, we could replicate the findings of the original study. We could also demonstrate that activity patterns preceding the time-point of the conscious decision became increasingly similar with increasing temporal proximity to the decision. Our behavioural data and questionnaire results further supported that no conscious processes biased the decisions. Thus, early predictive activity patterns are attributable to unconscious components of evolving intentions.

Comparable to the original study [30] subjects' intentions could be read out approximately seven seconds before they became conscious. Given the haemodynamic delay, it is likely that this reflects neural processes that occurred even earlier by a few seconds. The site of information encoding was found to be left frontopolar cortex, also referred to as the rostral lateral prefrontal cortex or the anterior prefrontal cortex, and approximating to the most anterior part of Brodmann area 10 [37]–[39]. The same region was identified in the original study but in the opposite hemisphere. In the present study, we optimized the slice positioning to minimize distortion effects and signal dropouts, which are a common problem due to the proximity to frontal sinuses, especially at higher field strength. Since the analysis only included voxels that were present in all subjects, residual dropout in individual subjects could have led the exclusion of more informative voxels. Hence, our results might underestimate the extent of the decision-related region. No information about the subjects' intentions was found after the decision was made, which is also in line with the original findings that after the time of the decision, information was only encoded in primary motor cortex and pre-motor cortex [30]. These areas were not covered in the present study. Due to the optimized slice positioning in the present study, the precuneus/posterior cingulate cortex, which additionally encoded early decision-related signals in the original study [30], was also not covered. As demonstrated before, the procedure used in both studies ensured that decoding could not be explained by activity related to the previous trial. As in our previous work [29], [30] we used a Finite Impulse Response (FIR) model, which is designed to separate effects of the current trial from the previous and the following trial. This method is highly efficient as long as both types of responses are roughly equally frequent, as here. Importantly, subjects self-paced their decisions, ensuring that the intervals between trails were variable, which makes the estimation of the FIR model even more robust to carry-over effects. Second, the time delay between the onset of predictive information in frontopolar cortex and the end of the previous trial was on average ∼15 seconds, and thus far beyond the relaxation time of the haemodynamic response. The average trial duration in the present study was even longer than in the original experiment, thus making it less likely that spill-over effects from the previous trials might have occurred. For the earliest time points in a trial we find no predictive information (as would have been expected if carry-over effects occurred). However, as time continues, we begin to see information. Third, the temporal resolution was also improved (1.5 s per time-bin compared to 2 s per time-bin in the original study), further validating the original findings. Fourth, data from one trial cannot be used to predict the trial preceding or following it. Fifth, carry-over effects are further unlikely because the distribution of response sequences resembled an exponential distribution, as expected for random behaviour (Fig. 5A). Although we do not believe and do not claim that our subjects produced perfectly random sequences, our behavioral results suggest that subjects made spontaneous decisions. This is probably because we did not ask subjects to balance their decisions.

Interestingly, we observed an increase in similarity between patterns with increasing temporal proximity to the conscious decision. This increase in correlation was mirrored by the increase in information content about the decision outcome. Thus, one possible explanation for this finding is that during the unconscious phase of intention-formation, the patterns slowly “evolved” towards the final conscious decision, comparable to a diffusion process postulated for fast, stimulus-driven decisions [40]. This hypothesis states that once a threshold is crossed (a certain pattern is stable enough), a conscious decision is made and activation patterns lose their predictive power afterwards. The remaining (but reduced) pattern stability might be explained by the dependence of sequentially acquired brain scans. Although there was some tendency for patterns to remain stable for a few seconds after the decision, there was no decodable information at these post-decision time periods. Similarly, patterns during the initial phase of the following trial were not informative. It was only later, closer to the next decision in the next trial, that we again observed a slow increase of pattern similarity and information encoding. This again speaks against carry-over effects from the previous trial. Our detailed behavioural analysis confirmed that subjects did not use any systematic thoughts to consciously prepare their decision ahead of time. They acted as instructed and were spontaneous. In support of these findings, Soon et al. [30] did not observe any encoding of the chosen movement in motor cortices before the decision; however, this could be expected when subjects pre-plan a motor response [29]. Here this analysis was not possible due to the restriction of coverage to PFC which was necessary to achieve a higher spatial resolution. We thus conclude that the early informative spatial activation patterns in frontopolar cortex were related to unconscious components of the intention. This again supports the hypothesis of a slow unconscious diffusion process towards a “prototypical” pattern in FPC, which is then related to the conscious decision. It might be surprising that decision-related information is encoded in the brain several seconds before the decision becomes conscious, given that the task was rather simple. One possibility is that random activity directly preceding the decision might bias the decision outcome, as suggested for short time periods [41]. This, however, is less likely for such long periods as observed here. Our study might have facilitated the detection of very early information by encouraging subjects to relax and refrain from decision-related thoughts as well as by instructing subjects to self-pace their decisions. By doing so, unlike most other studies, our experiment was uniquely suited to investigate the early evolution of intentions. Another possibility is that, even though we have good evidence that our subjects' behaviour was spontaneous, there might still have been some urge to respond regularly to a certain degree, which might only become detectable in longer behavioural sequences than produced here. Such a bias, even though outside subjects' awareness, could potentially contribute to the build-up of early brain activation patterns. It is important to note that any temporal autocorrelation in the signals could cause a correlation between choices in successive trials, even without a conscious, deliberative link. Such autocorrelation might be considered a very basic form of memory, but our conclusion that choices can be predicted before awareness would remain unchanged. Please also note that our study cannot provide evidence for a causal relationship between the activation in frontopolar cortex and the decision, e.g. because fMRI measures neural decision-related processes only indirectly and prediction is far from perfect. Our study also did not address the question of whether inter-individual differences, e.g. in intelligence, imaginativeness or other cognitive functions, might moderate the time-scale in which a build-up of decision-related activation patterns can be observed. Thus, one intriguing question for future research will be whether the onset of unconscious decision formation that can be decoded from brain activity might in turn be predictable by some core cognitive ability.

The present study supports the hypothesis that prefrontal cortex is a core region for free decisions. Presently, it is believed that the anterior prefrontal cortex lies at the top of a hierarchically organized prefrontal functional architecture. Prefrontal cortex represents sensory input information in its most abstract form and guides cognitive control [42]. It maintains the abstract representation of a desired act, together with context-relevant information such as environmental context, task-rules, motivation and potential outcomes [29], [43]–[45]; the motor plan for the execution of this act is prepared in premotor areas; this is broken down into co-ordinated recruitment of single motor units in primary motor cortex [46]. Medial prefrontal cortex might additionally contribute to action planning by processing self-related information [47], in this case, one's intentions; it was also found to encode freely chosen decisions during a delay [27].

Of the different regions in prefrontal cortex, however, evidence from cytoarchitectural studies suggests that frontopolar cortex has the necessary architecture to support the highest level of processing within prefrontal cortex. First, it has the greatest number of dentritic spines per cell, and overall spine density is higher than for all other areas of prefrontal cortex. Furthermore, it is the only supramodal area that is connected solely with other supramodal areas, has less laminar differentiation compared to other prefrontal areas, and its connections within PFC point towards a hierarchically high level of processing [20], [48], [49]. Given these properties, frontopolar cortex is an optimal candidate for the representation of the most abstract contents [20]. Current hypotheses about the function of this region are based mainly on functional imaging studies (as this region is markedly smaller and difficult to access in primate electrophysiology). Presently, the cognitive processes in which frontopolar cortex has been implicated include: processing of internal states [50], modulation of episodic memory retrieval [51], [52], prospective memory [53], relational reasoning [54], [55], the integration of cognitive processes [39] and cognitive branching [19]. In this last model, frontopolar cortex was suggested to control long-term plans and to generate new cognitive sequences [19]. This is supported by recent findings showing that frontopolar cortex also tracks the advantage of alternative action plans and might initiate switching [56]. Burgess et al. [57] proposed that the type of processing in frontopolar cortex is determined by the context, allowing either stimulus-oriented (i.e., pertaining to the external environment) or stimulus-independent (i.e., pertaining to the internally generated representations) processing to occur. These theories are in line with a role of frontopolar cortex in the generation of free decision as demonstrated by Soon et al. [30] and the present study.

One possibility is that neurons in frontopolar cortex could be tuned to different decision outcomes, while having the capacity to be flexibly re-coded depending on task demands as previously suggested for prefrontal cortex [58], [59]. This mechanism would also allow different types of intentions to be encoded without the need for hardwiring of single neurons to any single intention. Most abstract intentions are closely linked to some motor action anyway and might therefore be represented in a similar manner. Additionally, it has been proposed that evolutionarily newer functions, such as cultural inventions, could make use of already existing neural structures evolved for more basic but similar functions [60]. Future studies should address this question by investigating the encoding of more abstract intentions, such as performing mathematical calculations, and the functional organization of the architecture that gives rise to them. One advantage of using fMRI for this purpose compared to readiness potentials (RPs) as in the classical Libet study [5] is that fMRI allows the investigation of the whole brain at high spatial resolution. The temporal resolution of fMRI compared to EEG is low. On the other hand, it offers the possibility to assess activation in a multitude of brain regions at early stages of the decision process. Please note, however, that even though fMRI is uniquely suited to investigate large-scale changes in cortical networks, and multivariate pattern classification additionally increases the sensitivity of the analysis [24], [25], the precise mechanism behind decision formation can surely not be revealed by fMRI alone.

In summary, we could replicate the finding of Soon et al. [30] that motor intentions were encoded in frontopolar cortex up to seven seconds before participants were aware of their decisions. Using ultra-high field fMRI on a 7 Tesla scanner, we could show that these patterns became more stable with increasing temporal proximity to the conscious decision. These findings support the conclusion that frontopolar cortex is part of a network of brain regions that shape conscious decisions long before they reach conscious awareness.
